# *Mansonella perstans*, *Onchocerca volvulus* and *Strongyloides stercoralis* infections in rural populations in central and southern Togo

**DOI:** 10.1016/j.parepi.2018.03.001

**Published:** 2018-03-13

**Authors:** Francois Korbmacher, Kossi Komlan, Richard G. Gantin, Wiyao P. Poutouli, Koffi Padjoudoum, Potchoziou Karabou, Peter T. Soboslay, Carsten Köhler

**Affiliations:** a National Institute of Hygiene, Onchocerciasis Reference Laboratory, Sokodé, Togo; b Institute for Tropical Medicine, University Clinics of Tübingen, Germany; c Faculté de Sciences, Université de Lomé, Lomé, Togo; d National Onchocerciasis Control Program, Kara, Togo

**Keywords:** *Onchocerca volvulus*, *Mansonella perstans*, *Strongyloides stercoralis*, Onchocerciasis, Mansonelliasis, Strongyloidiasis, Togo, Prevalence

## Abstract

**Background:**

*Mansonella perstans*, *Onchocerca volvulus* and *Strongyloides stercoralis* are widespread helminth parasites in the tropics. Their distribution remains difficult to determine as it may change during national disease control programs and with regional mass drug administration (MDA). Epidemiological surveys are of importance to evaluate the geographical distribution of these helminth parasites and the diseases they may cause, however, up to date epidemiological evaluations on *M. perstans* and *S. stercoralis* in Togo are rare, and surveys on *O. volvulus* are important especially under the aspect of MDA of ivermectin which is performed since decades.

**Methods:**

Dry blood samples (*n* = 924) were collected from rural populations in the Régions Central and Plateaux in Togo, and analyzed by parasite-specific real-time PCR and ELISA techniques.

**Results:**

Dry blood samples from 733 persons where investigated by real-time PCR tested for DNA of blood-circulating *M. perstans* microfilaria, and a prevalence of 14.9% was detected. Distinct differences were observed between genders, positivity was higher in men increasing with age, and prevalence was highest in the Région Plateaux in Togo. IgG4 responses to *O. volvulus* antigen (OvAg) were studied in 924 persons and 59% were found positive. The distribution of parasite infestation between age and gender groups was higher in men increasing with age, and regional differences were detected being highest in the Région Plateaux. The diagnostic approach disclosed 64,5% positive IgG4 responses to *S. stercoralis* infective third-stage larvae-specific antigen (SsL3Ag) in the surveyed regions. Antigen cross reactivity of SsL3Ag with parasite co-infections may limit the calculated prevalence. Singly IgG4 positive for SsL3Ag were 13.9%, doubly positive for OvAg and SsL3Ag were 35.5% and triply positive for *M. perstans*, *O. volvulus* and *S. stercoralis* were 9.9%.

**Conclusions:**

Mansonelliasis, onchocerciasis and strongyloidiasis remain prevalent in the surveyed regions, yet with local differences. Our observations suggest that transmission of *M. perstans*, *O. volvulus and S. stercoralis* may be ongoing. The degree of positive test results in the examined rural communities advocate for the continuation of MDA with ivermectin and albendazole, and further investigations should address the intensity of transmission of these parasites.

## 1 Introduction

*Mansonella perstans*, *Onchocerca volvulus* and *Strongyloides stercoralis* are widespread helminth parasites in the tropics. *M. perstans* is endemic in central (Noireau et al., 1990) and western (Bassene et al., 2015; Debrah et al., 2017) Africa, as well as in Central and Latin America (Simonsen et al., 2011, Carvalho and Silva, 2014). The adult filariae of *M. perstans* are found in the connective tissue of the serous body cavities and the microfilariae which are released by gravid female filariae circulate in the peripheral blood (Asio et al., 2009b). The transmission of *M. perstans* is by *Culicoides* spp. (midges, Ceratopogonidae), a cosmopolitan genus with species that transmit besides *Mansonella* spp. also viral and protozoan pathogens (Sharp, 1928; Mellor et al., 2000; Rebêlo et al., 2016; Svobodová et al., 2017). Unclear symptoms of an infection and the lack of diagnosis lead to inaccurate epidemiological data on mansonelliasis (Simonsen et al., 2011), and the prevalence in endemic countries is variable due to bio-ecological zones (Wanji et al., 2016).

*Onchocerca volvulus* is taxonomically classified as *Mansonella* spp. into the nematode family of Onchocercidae. In the human host the adult filariae of *O. volvulus* reside in nodular fibrous granulomas formed as a result of the host immune defence. In those nodules female gravid *O. volvulus* release larvae, the microfilariae (Mf), which migrate in subcutaneous tissues causing dermal irritations, inflammation and skin lesions. When entering the eyes Mf will damage corneal and retinal tissues which ultimately may result in blindness after years of infection. In West Africa *O. volvulus* transmission and human infection occurs with the blood meal of black fly species of the *Simulium damnosum s.l.* genus which breed in fast-flowing rivers, hence the name river blindness. An estimated 37.2 million are infected with *O. volvulus,* about 1 million are blinded by onchocerciasis, and 99% of the infected live in Africa (Tekle et al., 2016).

*Strongyloides stercoralis* infection is initially by third-stage larvae (L3) which penetrate the skin and migrate through blood vessels and lungs to finally mature in the small intestine where gravid female worm release first-stage larvae. Those L1 larvae are either passed with the faeces, or L1 will auto-infect the human host. Such continuous auto-infections and parthenogenic reproduction of female *S. stercoralis* will establish in the human host persistent and chronic infections. Strongyloidiasis is found worldwide, with higher prevalence in the tropics and subtropics with an estimated 100 million infected (Rodrigues et al., 2007).

In Togo epidemiological evaluations on *M. perstans* are rare, surveys on *O. volvulus* are important especially under the aspect of mass drug administration (MDA) of ivermectin which is performed since decades (Banla et al., 2014), and data on the prevalence of *S. stercoralis* infection are not available. We have accomplished immune-epidemiological and molecular surveys on the prevalence of *M. perstans*, *O. volvulus* and *S. stercoralis* in rural areas in central and southern Togo. Such evaluations provide specific data on the regional distribution of mansonelliasis, onchocerciasis and strongyloidiasis, and such mapping is indispensable for control efforts and interventions.

## 2 Material and methods

### 2.1 Study area and population

The survey participants are resident in the villages of Tsokple (7°23′57.6″N 1°06′36.0″E), Kpati-Cope (7°22′29.1″N 1°07′47.3″E), Igbowou-Amou (7°23′02.0″N 1°06′29.3″E), Atinkpassa (7°22′07.9″N 1°32′04.8″E), Amouta (7°33′27.6″N 0°56′26.4″E) and Tutu-Zionou (7°05′20.5″N 0°54′42.8″E) in the Région Plateaux in Togo. The villages Sagbadai (9°04′06.1″N 1°04′17.9″E), Bouzalo (9°06′05.4″N 1°02′37.8″E) and Kéméni (9°14′03.7″N 1°14′36.4″E) are located in the Région Centrale. All villages are under surveillance of the Togolese National Onchocerciasis Control Program (NOCP). The protocol of the study was reviewed and approved by the Togolese Bioethics Committee for Research in Health (Comité de Bioéthique pour la Recherche en Santé (CBRS); Avis #015/2012/CBRS, Document #2804/2012/MS/CAB/DGS/DPLET/CBRS/16.November 2012, Document #013/2015/CBRS/3.Septembre 2015), and study authorization and approval was granted by the Ministry of Health of Togo (Authorization; Document #338/2015/MSPS/CAB/SG). Consent from each study participant was documented and confirmed by signature, and consent for study participation by those <18 years of age was given verbally by each participant, and written consent and approval for participation for those <18 years was always obtained by the parents or the accompanying responsible adults. For correct and complete understanding explanations were always given in the local language. Before each follow up survey, approval was obtained from the appropriate regional (Direction Régional de la Santé de la Population) and district-level (Direction Préfectural de la Santé) health authorities.

All specimens used in this study were collected from study participants who provided written informed consent. The minimum age of participants was ≥10 years in the Région Centrale and ≥5 years in the Région Plateaux. For the Région Plateaux, the dry blood spot sample collections were accompanied in parallel with the collection of skin biopsies to determine the prevalence of *O. volvulus* microfilariae. In the central region DBS were collected without skin biopsies. In the Région Centrale and Plateaux the village populations received since several years annually by means of mass drug administration (MDA) ivermectin (≥5 years, 150 μg/kg). In addition, t**he National** L**ymphatic Filariasis** Elimination Program (NPELF) in Togo has extended the distribution system established by the onchocerciasis program through the co-administration of albendazole with ivermectin (Sodahlon et al., 2013).

### 2.2 Sampling of dry blood spots

The blood samples were collected by medical assistants and conserved as dry blood spots on protein saver cards type 903™ (Whatman, USA). The samples were coded immediately, air-dried, sealed air tight and stored at +8 °C. The DBS samples served for DNA extraction as well as for antibody elution for ELISA purposes.

### 2.3 Skin biopsies

With corneo scleral punches one skin biopsy was taken from the upper dermis (2–3 mm in size) on the left and right iliac crest of the hip to sum up for a total of two samples. Skin biopsies were immediately incubated in physiological saline solution on segmented glass slides and after 30 min the slides were examined under a microscope for *O. volvulus* microfilariae (Mf) emerging from the biopsies and the Mf numbers were counted.

### 2.4 DNA extraction from dry blood spots

From the protein cards completely dry blood circles (diameter approx. 6.5 mm) were cut out with a punching scissor. From these dry blood circles the DNA was extracted by using the QIAamp DNA Mini Kit (QIAGEN, Netherlands) according to the recommended dry blood spot protocol.

### 2.5 Real-time PCR

The detection of parasite DNA was carried out by real-time PCR (rtPCR) using the PCR rotor gene cycler RG3000 (Corbett Research/Corbett Life Science, QIAGEN, Netherlands). The primers for the respective parasites are shown with their PCR conditions in Table 1. The primer and probe selection was accomplished by using the online software Primer3 (http://bioinfo.ut.ee/primer3-0.4.0/). The real-time PCR primer pairs, probes and test conditions used for the detection of *Mansonella perstans* (Mp) were: Mp-primer-fwd 5′-CTGCGGAAGGATCATTAA-3′ (Tm 51.4 °C); Mp-primer rev 5′-TGCATGTTGCTAAATAAAAGTG-3′ (Tm 52.8 °C); Mp-probe 5′-FAM-CGAGCTTCCAAACAAATACATAATAAC-TAM-3′ (Tm 58.9 °C); The rtPCR conditions were 50 °C/2 min, 95 °C/10 min, [95 °C/15 sec, 53 °C/1 min] × 45.

**Table 1:** Summary of *Mansonella perstans* DNA detection in dry blood samples by means of parasite-specific real-time PCR. The examined participants are listed by region, village, gender and age groups. The number of DNA-positive samples in the examined groups and the prevalence of positivity in % are shown.

Region	Village	Male Pos/Neg (% Pos)	Female Pos/Neg (% Pos)	<15y Pos/Neg (% Pos)	16–25y Pos/Neg (% Pos)	26–35y Pos/Neg (% Pos)	***M.perstans* DNA Pos/Neg (% Pos)**
Centrale	Sagbadai	2/41(4,9)	5/59(8,5)	0/19(0)	2/16(0)	2/14(14,3)	**7/100(7,0)**
	Bouzalo	13/52(25,0)	6/48(12,5)	0/15(0)	0/10(0)	4/20(20,0)	**19/100(19,0)**
	Kéméni	1/96(1,0)	0/101(0)	0/64(0)	0/32(0)	0/12(0)	**1/197(0,5)**
	**TOTAL**	**16/189(8,5)**	**11/208(5,3)**	**0/98(0)**	**2/58(3,4)**	**6/49(12,2)**	**27/397(6,8)**
Plateaux	Tsokple	9/37(24,3)	9/52(17,3)	4/29(13,8)	3/17(17,6)	4/20(20,0)	**18/89(20,2)**
	Kpati Cope	13/40(32,5)	13/43(30,2)	2/22(9,1)	2/8(25,0)	6/18(33,3)	**26/83(31,3)**
	Igbowou-Amou	4/29(13,8)	5/52(9,6)	0/17(0)	0/13(0)	1/10(10,0)	**9/81(11,1)**
	Atinkpassa	16/38(42,1)	13/45(28,9)	3/20(15)	5/16(31,3)	7/22(31,8)	**29/83(34,9)**
	**TOTAL**	**42/144(29,2)**	**40/192(20,8)**	**10/88(11,4)**	**10/53(18,9)**	**18/64(28,1)**	82/336(24,4**)
**TOTAL**		58/333(17,4*)	**51/400(12,8)**	**10/186(5,4^$^)**	**12/111(10,8)**	**63/326(19,3)**	**109/733(14,9)**

**p < 0.0001 Region Centrale *vs.* Plateaux; *p* = 0.05 Male *vs*. Female; $ *p* = 0.0001 Group <15 y *vs*. Group 26–35y.

### 2.6 *Onchocerca volvulus* and *Strongyloides stercoralis* antigen

For the OvAg-IgG4 ELISA an adult worm antigen extract from male and female *O. volvulus* was used. The preparation of the *O. volvulus* antigen (OvAg) was accomplished as previously described by Mai et al., 2007 and Lechner et al., 2012. Briefly, adult worms were isolated as described (Schulz-Key et al., 1977) and then washed in phosphate-buffered saline (PBS), transferred into a Ten-Broek tissue grinder and then homogenized extensively on ice. The homogenate was then sonicated twice (30% intensity) for 3 min on ice and centrifuged at 16,000*g* for 30 min at 4 °C. The *S. stercoralis* (SsAg) antigen was prepared from third-stage larvae (L3, *n* = 8 × 10^5^) of *S. stercoralis* which were kindly made available by Prof. James B. Lok (Pennsylvania State University, USA). The L3 larvae were pooled in 2 ml of PBS, homogenized on ice for 30 min with a Ten-Boek tissue grinder, and then pulse ultra-sonicated (30% intensity) on ice for 10 min. This homogenate was then centrifuged for 15 min at 16,000*g*, the supernatant collected and the protein concentration of this PBS-soluble *S. stercoralis* L3 antigen extract (SsL3Ag) determined (BCA Protein Assay, PIERCE, Rockford, USA). The OvAg and SsL3Ag preparations were further used for the antigen-specific IgG4 ELISAs.

### 2.7 Antibody-ELISA

From the protein saver cards blood circles (diameter 6.5 mm) were cut out with a punching device. The elution of antibodies from the cards was with 200 μl of PBS containing 0.05% Tween20 and 5% bovine serum albumin (BSA, Fraction V, Sigma-Aldrich Co., USA) for 2 days at 4 °C in Nunc **96 DeepWell polypropylene plates (Sigma Aldrich, Merck, P6866).** For the OvAg-IgG4 ELISA an adult worm antigen extract from male and female *O. volvulus* was applied to measure serological IgG4 responses. The sensitivity of the IgG4-directed *O. volvulus* adult worm antigen (OvAg)-specific ELISA was determined with a contingency analysis and calculated against the results of skin biopsies. The calculated sensitivity of the IgG4-OvAg-ELISA was 93.1%. Micro titer plates (Costar 3690, half area) were coated with OvAg (conc. 5 μg/ml) or SsL3Ag (conc. 5 μg/ml) in PBS pH 7.4 overnight, then the coating antigen solutions were discarded, and plates were blocked with PBS-Tween20® containing 5% fetal bovine serum at room temperature for 1.5 h. Thereafter, plates were washed with PBS-Tween20® (Sigma P3563), eluted blood samples were added without dilution and plates incubated at 37 °C for 2 h. After using PBS-Tween20® to wash an anti-human IgG4, horse radish peroxidase conjugated monoclonal antibody (Thermo Fisher Scientific, no.A10654) was added (dilution 1:500) for 1.5 h, then plates were washed as above and TMB substrate (Thermo Scientific 34021) was added. Plates were incubated at room temperature for 15 min and the reaction was stopped with 50 μl of 0.5 M sulfuric acid (ROTH, K027.1), and optical densities were measured at 450 nm with a micro plate reader (EL808, BioTex Instruments).

### 2.8 Data analysis

The collected data were analyzed using statistic software SAS JMP 11.1.1. The IgG4 responses (optical density, OD) of samples were determined in duplicate. First, from the brut ODs the background values (mean OD of blank wells of the ELISA plate) were subtracted to obtain the net OD. The negative/positive threshold values (cut off) were determined for each plate by calculating the mean IgG4 ODs of 10 samples from *O. volvulus* Mf-negative (MW-NEG-OD) participants (Région Plateaux) and then 4× of their standard deviation was added to the MW-NEG-OD. The sensitivity of the OvAg-specific IgG4 ELISA was determined with a contingency analysis; here, the OvAg-specific IgG4 responses and the *O. volvulus* Mf-positive and negative skin biopsy results were applied to determine the sensitivity of the ELISA. The negative/positive threshold values (cut off) for the OvAg-IgG4 and the SsL3Ag-specific IgG4 responses from the Région Central were calculated as described above; *i.e.*, for each ELISA plate the mean values of the lowest IgG4 ODs of 8 blood samples (MW-NEG-OD) were determined and then 4× of their standard deviation was added.

## 3 Results

### 3.1 *Mansonella perstans* specific DNA in blood samples

In Table 1 the detection of *M. perstans* DNA in dry blood samples by means of parasite-specific real-time PCR is detailed by region, village, gender and age groups; the number of DNA-positive samples and % positivity is shown. The overall prevalence for *M. perstans* DNA positivity was 14.9%. The difference between the Région Centrale (6.8% positive) and Région Plateaux (24.4% positive, Fisher-Exact-Test: *p* < 0.0001) is particularly noticeable. Men (17.4%) were more often positive than women (12.8%, Fisher-Exact test: *p* = 0.05). The age group under 15 years (5.4%) was less often affected than the age group between 16 and 25 years (10.8%, Fisher-Exact test: *p* = 0.07) and the 26–35 years age group (19.3%, Fisher-Exact Test: *p* = 0.0001). The lowest prevalence for *M. perstans* was found in the village of Kéméni/Région Centrale with 0.5%, and the highest was in Atinkpassa/Région Plateaux with 34.9%. In the *M. perstans*-positive patients the ct-values of the parasite-specific rt-PCR decreased with increasing age (Spearman rank co-efficient: *ρ* = −0.263; *p* = 0.0055) which indicated that *M. perstans* DNA-levels steadily enhanced over age (Fig. 2).

### 3.2 *Onchocerca volvulus* microfilariae (mf) in skin biopsies

Skin biopsies were collected from the participants in the Region Plateaux, and 5,3% (29 mf-positive/528 examined) of them were positive for microfilaria of *O. volvulus*. In Tsokle 12.4% (12 mf-pos/97 examined), Igbowou-Amou 6.9% (6/87), Kpati Cope 6.7% (6/89), Atinkpassa 3.4% (3/88), Tutu Zionou 2.5% (2/79) and in Amouta 0% (0/88) were positive for mf of *O. volvulus*.

### 3.3 IgG4 responses to *Onchocerca volvulus* antigen (OvAg)

The sensitivity of the IgG4-directed *O. volvulus* adult worm antigen (OvAg)-specific ELISA against the results of skin biopsies was evaluated, and the calculated sensitivity of the IgG4-directed OvAg-specific ELISA was 93.1%. In Table 2 the OvAg-specific IgG4 responses are shown by region, village and separated by gender and age groups. The overall prevalence of IgG4 sero-positivity was 59.0% for OvAg. The inhabitants from villages in the Région Plateaux showed with 75.4% significantly more positive reactions than the village inhabitants from the Région Centrale (37.1%) (Fisher-Exact-Test: *p* < 0.0001). IgG4 responses to OvAg in men (61.4%) were more often positive than in women (56.8%) (Fisher-Exact test: *p* = 0.09). Between the two youngest age groups (<15 years, 49.3% seropositive and 15–25 years, 55.4% seropositive) IgG4 positivity did not differ (*p* = 0.28). In the older age groups, positive response were significant higher in the 25–35-year-olds (Fisher's Exact Test: *p* = 0.0002) and also in those above 35 years (Fisher Exact Test: *p* = 0.001) when compared with the youngest age group. The lowest sero-positivity with 32.1% was detected in the village of Kéméni/Région Central and the highest was with 85.2% in Amouta/Région Plateaux. The OvAg-specific IgG4 responses (OD) heightened with increasing age (Fig. 3) (Spearman rank co-efficient: *ρ* = 0.1633; *p* < 0.0001) indicating persisting *O. volvulus* infections and suggesting an accumulation of parasites over age.

**Table 2:** IgG4 antibody responses to *Onchocerca volvulus* antigen (OvAg). The results are listed by region, village, gender and age groups. The number of participants and positive responses in the examined group and the prevalence of positivity in % are shown.

Region	Village	Male Pos/Neg (% Pos)	Female Pos/Neg (% Pos)	<15y Pos/Neg (% Pos)	16–25y Pos/Neg (% Pos)	26–35y Pos/Neg (% Pos)	>35y Pos/Neg (% Pos)	**OvAg-IgG4 Pos/Neg (% Pos)**
Centrale	Sagbadai	17/41(41,5)	21/59(35,6)	8/19(42,1)	5/16(31,3)	7/14(50,0)	18/51(35,3)	**38/100(38,0)**
	Bouzalo	26/52(50,0)	19/48(39,6)	5/15(33,3)	2/10(20,0)	10/20(50,0)	28/55(50,9)	**45/100(45,0)**
	Kéméni	36/95(37,9)	28/101(27,7)	18/63(28,6)	13/32(40,6)	7/12(58,3)	26/89(29,2)	**64/196(32,7)**
	**TOTAL**	**79/188(42,0)**	**68/208(32,7)**	**31/97(32,0)**	**20/58(34,5)**	**24/46(52,2)**	**72/195(36,9)**	**147/396(37,1)**
Plateaux	Tsokple	26/41(63,4)	33/56(58,9)	15/31(48,4)	11/19(57,9)	10/18(55,6)	23/29(79,3)	**59/97(60,8)**
	Kpati Cope	38/45(84,4)	34/44(77,3)	15/25(60,0)	7/8(87,5)	15/19(78,9)	35/37(94,6)	**72/89(80,9)**
	Igbowou-Amou	24/33(72,7)	40/54(74,1)	9/17(52,9)	7/12(58,3)	4/6(66,7)	44/52(84,6)	**64/87(73,6)**
	Atinkpassa	30/42(71,4)	39/46(84,7)	14/20(70,0)	14/18(77,8)	20/24(83,3)	21/26(80,8)	**69/88(78,4)**
	Amouta	37/44(84,1)	38/44(86,4)	15/18(83,3)	10/14(71,4)	14/15(93,3)	36/41(87,8)	**75/88(85,2)**
	Tutu Zionou	32/40(80,0)	27/39(69,2)	11/15(73,3)	8/10(80,0)	10/14(71,4)	30/41(73,2)	**59/79(74,8)**
	**TOTAL**	**187/245(76,3)**	**211/283(74,6)**	**79/126(62,7)**	**57/81(70,4)**	**73/95(76,8)**	**189/226(83,6)**	398/528(75,4**)
**TOTAL**		**266/433(61,4)**	**279/491(56,8)**	**110/223(49,3)**	**77/139(55,4)**	97/141(68,8*)	**261/421(62,0^$^)**	**545/924(59,0)**

**p < 0.0001 Region Centrale vs. Plateaux; *p* = 0.0002 Group 26–35 y *vs*. Group <15 y; $ *p* = 0.001 Group >35 y *vs*. Group <15 y.

### 3.4 IgG4 responses to *Strongyloides stercoralis* third-stage larvae (L3) antigen (SsL3Ag)

In Table 3 the IgG4 responses to SsL3Ag are shown separated by region, village, gender and age groups. The overall prevalence of sero-positivity was 64.5% for *S. stercoralis.* In the Région Plateaux positive IgG4 responses with 71.2% to SsL3Ag were observed more often than in the Région Centrale with 55.5% (Fisher-Exact Test: *p* < 0.0001). Positive IgG4 responses were similar in men (67.1%) and women (62.2%) (Fisher-Exact test: *p* = 0.07). In the two youngest age groups (up to 15 years: 57.5%, 16–25 years: 61.9%) the sero-positivity did not differ (Fisher-Exact-Test: *p* = 0.44). IgG4 responses to SsL3Ag in the 26–35 years group (72.1%, Fisher-Exact-Test: *p* = 0.003) and in those above 35 years were found more often (66.5%, Fisher-Exact-Test: *p* = 0.02) than in those under 15 years of age. The lowest sero-prevalence was observed in the village of Kéméni/Région Centrale (45.4%) and the highest in Atinkpassa/Région Plateaux (84.1%). The SsL3Ag-specific IgG4 responses (OD) did not correlate with the age of the participants (Fig. 4, upper graph) but they correlated positive (Spearman rank co-efficient: *ρ* = 0.6985; *p* < 0.0001) with the OvAg-specific IgG4 reactivity (Fig. 4, lower graph).

**Table 3:** The IgG4 antibody responses to *Strongyloides stercoralis* L3 antigen (SsL3Ag). The results are listed by region, village, gender and age groups. The number of participants and positive responses in the examined group and the prevalence of positivity in % are shown.

Region	Village	Male Pos/Neg (% Pos)	Female Pos/Neg (% Pos)	<15y Pos/Neg (% Pos)	16–25y Pos/Neg (% Pos)	26–35y Pos/Neg (% Pos)	>35y Pos/Neg (% Pos)	**IgG4-SsL3Ag Pos/Neg (%Pos)**
Centrale	Sagbadai	29/41(70,7)	40/58(69,0)	13/19(68,4)	10/16(62,5)	9/13(69,2)	37/51(72,5)	**69/99(69,7)**
	Bouzalo	33/52(63,5)	28/48(58,3)	7/15(46,7)	4/10(40,0)	13/20(65)	37/55(67,3)	**61/100(61,0)**
	Kéméni	46/93(49,5)	42/101(41,6)	23/61(37,7)	16/32(50,0)	7/12(58,3)	42/89(47,2)	**88/194(45,4)**
	**TOTAL**	**108/186(58,1)**	**110/207(53,1)**	**43/95(45,3)**	**30/58(51,7)**	**29/45(64,4)**	**116/195(59,5)**	**218/393(55,5)**
Plateaux	Tsokple	35/41(85,4)	36/56(64,3)	27/31(87,1)	12/19(63,2)	12/18(66,7)	20/29(69,0)	**71/97(73,2)**
	Kpati Cope	31/45(68,9)	28/44(63,6)	14/25(56,0)	7/8(87,5)	12/19(63,2)	26/37(70,3)	**59/89(66,3)**
	Igbowou-Amou	14/33(42,4)	28/54(51,9)	2/17(11,8)	4/12(33,3)	3/6(50,0)	33/52(63,5)	**42/87(48,3)**
	Atinkpassa	34/42(81,0)	40/46(87,0)	16/20(80,0)	15/18(83,3)	22/24(91,7)	21/26(80,8)	**74/88(84,1)**
	Amouta	35/44(79,5)	35/44(79,5)	14/18(77,8)	10/14(71,4)	13/15(86,7)	33/41(80,5)	**70/88(79,5)**
	Tutu Zionou	32/40(80,0)	28/39(71,8)	11/15(73,3)	8/10(80,0)	10/13(76,9)	31/41(75,6)	**60/79(75,9)**
	**TOTAL**	**181/245(73,9)**	**195/283(68,9)**	**84/126(66,7)**	**56/81(69,1)**	**72/95(75,8)**	**164/226(72,6)**	376/528(71,2**)
**TOTAL**		**289/431(67,1)**	**305/490(62,2)**	**127/221(57,5)**	**86/139(61,9)**	101/140(72,1*)	280/421(66,5*)	**594/921(64,5)**

**p < 0.0001 Region Centrale *vs*. Plateaux; *p < 0.05 Groups 26–35 y and >35 y *vs*. Group <15 y.

### 3.5 Parasite co-infections in participants

In Table 4 single, double and triple infections with *Strongyloides stercoralis, Onchocerca volvulus* and *Mansonella perstans* in the study participants are shown. Singly positive for *M. perstans* (M.p.), *O. volvulus* (O.v.) or *S. stercoralis* (S.s.) were 2.1%, 7.0% and 13.9% of the participants, respectively. Doubly positive for *O. volvulus* and *M. perstans* were 1.2%, for *S. stercoralis* and *M. perstans* 1.8%, and for *O. volvulus* and *S. stercoralis* 35.4% were detected IgG4 positive. Of note, positive IgG4 responses to SsL3Ag and DNA-positive for *M. perstans* were 15.6% of the participants while being negative for IgG4 to *O. volvulus* antigen (OvAg). Triply positive for M.p., O.v. and S.s. were 9.9%. In 28.8% of the participants neither positive IgG4 responses to OvAg and SsL3Ag nor *M. perstans*-specific DNA were found.

**Table 4:** Single, double and triple infections with *Strongyloides stercoralis, Onchocerca volvulus* and *Mansonella perstans* in the study participants as determined by real-time PCR for *M. perstans* (M.p.), and by parasite antigen-specific ELISA for *O. volvulus* (OvAg) and *S. stercoralis* (SsL3Ag)*.* From all examined participants (*n* = 729) the test results for the respective infections (−/+), the number of cases (N) for the single, double and triple infection groups and their distribution (in % of total) are shown.

S.s. IgG4	O.v. IgG4	M.p. rtPCR	N (729)	%
–	–	–	210	28,8%
–	–	Mp+	15	2,1%
–	Ov+	–	51	7,0%
–	Ov+	Mp+	9	1,2%
Ss+	–	–	101	13,8%
Ss+	–	Mp+	13	1,8%
Ss+	Ov+	–	258	35,4%
Ss+	Ov+	Mp+	72	9,9%

## 4 Discussion

**Fig. 1:**
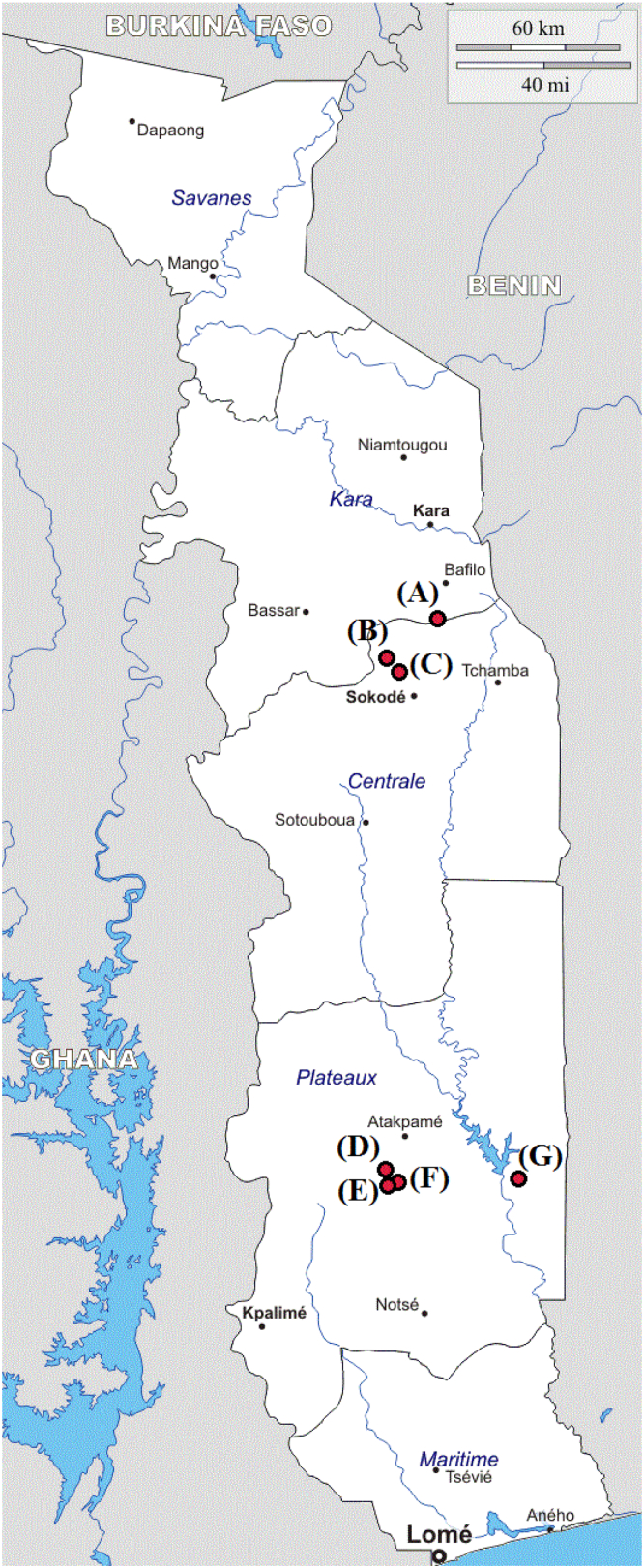
Map showing the locations of the surveyed villages Kéméni (A), Bouzalo (B) and Sagbadai (C) in the Région Central, and the villages Tsokple (D), Kpati Cope (E), Igbowou-Amou (F) and Atinkpasse (E) in the Région Plateaux in Togo. The regions were included into the vector control activities of the Onchocerciasis Control Program (OCP) in 1987 and in both regions, black fly vector control measures were supplemented in 1989 by mass drug administration (MDA) with ivermectin.

In all surveyed villages *M. perstans* DNA was detected in dry blood samples, and prevalence ranged from 0.5% (Kéméni, RC) to 34.9% (Atinkpassa, RP). Such local differences are known from mansonelliasis endemic areas and attributed to fluctuating environmental factors which influence the parasite and vector populations (Simonsen et al., 2011; Wanji et al., 2016). The surveyed villages in the Régions Plateaux and Central do not differ in altitude, climate and vegetation, and they are located in the arboreal savannah (Fig. 1) where agricultural activities dominate the activities of the village population. The higher mansonelliasis prevalence in males corresponds with earlier observations where a more intense exposure of man to infective *Culicoides* spp. vectors was mentioned as a possible cause, but also physiological parameters were suggested (Asio et al., 2009a, Noireau et al., 1989). The raise in prevalence with increasing age corresponded with previous studies (Jordan, 1955); the *M. perstans* Mf positive cases were older than the Mf negative, and the lowering ct-values in our rt-PCR with increasing age indicated that *M. perstans* Mf-levels steadily enhanced. Thus, repetitive exposure to *M. perstans* may not elicit protective immunity, and *M. perstans* infections will steadily accumulate.

**Fig. 2:**
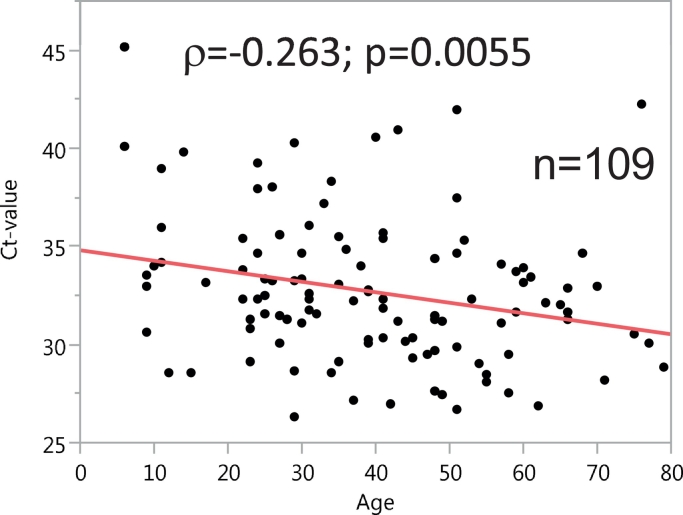
Correlation of the cycle threshold (ct) values of the *Mansonella perstans* specific real-time PCR for the detection of parasite DNA in blood with the age (in years) of the study participants.

**Fig. 3:**
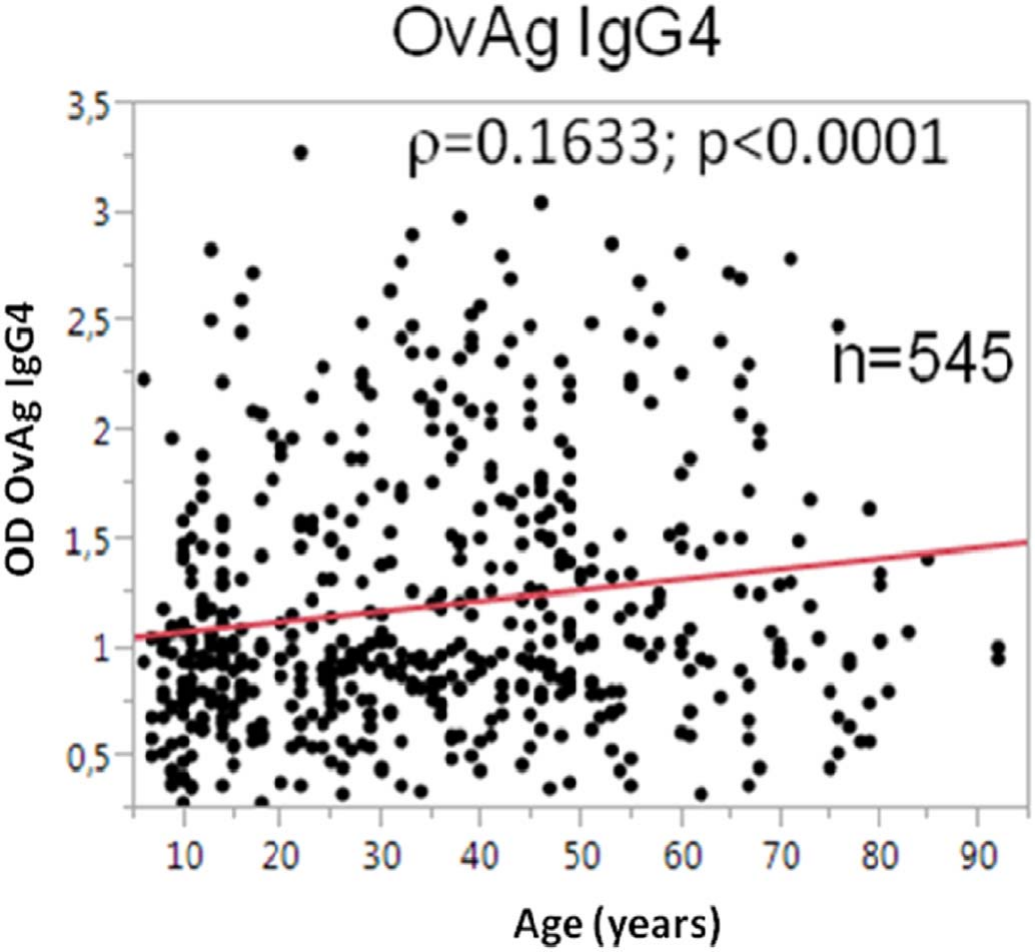
The IgG4 responses to *O. volvulus*-specific antigen (OvAg) in correlation with the age (in years) of the study participants.

**Fig. 4:**
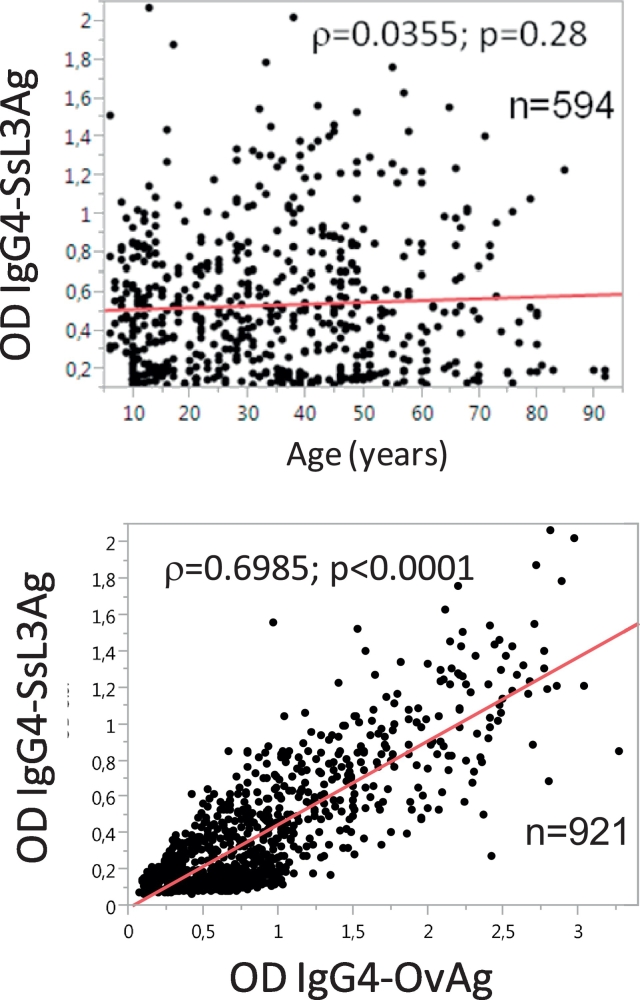
The IgG4 responses to *S. stercoralis* L3 antigen (SsL3Ag) in correlation with the age (in years) of the study participants (upper graph), and the correlation of the SsL3Ag-specific IgG4 and the OvAg-specific IgG4 reactivity (lower graph) (OD = optical densities).

For the detection of *M. perstans* we have chosen real-time PCR because *M. perstans*-specific antigens are not available. The *M. perstans*-specific rtPCR will detect blood-circulating microfilaria, and it will not respond to *O. volvulus* where microfilaria is skin-dwelling. The diagnostic approach by rtPCR allows the investigation of large sample numbers and DNA detection by PCR is more sensitive than by microscopy (Medeiros et al., 2015). Blood samples from mansonelliasis patients may contain few but also high Mf numbers per millilitre (Schulz-Key et al., 1993) and with the small volumes of blood (200 μl) collected on filter papers patients with low Mf densities may falsely have been declared infection-free. In addition, real-time PCR facilitates monitoring of intervention programs and allows species-specific detection of treatment failure following rounds of mass treatment (Verweij et al., 2009, Bregani et al., 2006, Basuni et al., 2011). In Togo, the Région Centrale and Plateaux were included into the control activities of the Onchocerciasis Control Program (OCP) in 1987 and in both areas the mass drug administration (MDA) of ivermectin is applied since 1989 until today. In both regions the *O. volvulus* microfilarial prevalence declined markedly, and onchocerciasis is considered as close to elimination (WHO, 2014, WHO, 2015, WHO, 2016, WHO, 2017). Onchocerciasis disease has largely been controlled with MDA of ivermectin (Sékétéli et al., 2002), and for the treatment of lymphatic filariasis the repeated administration of ivermectin together with albendazole is recommended (Ottesen, 2006; Taylor et al., 2010). While annually repeated ivermectin together with albendazole may have interrupted *W. bancrofti* parasite transmission in several districts in Togo (Sodahlon et al., 2013; Dorkenoo et al., 2015), the drug combination will not efficaciously eliminate *M. perstans* microfilariae and mansonelliasis will persist (Schulz-Key et al., 1993, Asio et al. 2009, Bregani et al., 2006).

Our serological ELISA to detect active *O. volvulus* infections was sensitive with 93%, and the antigen-specific IgG4 reactivity to OvAg can reflect the status and history of infection. In previous works we have applied the OvAg-IgG4-ELISA in surveys with onchocerciasis patients at distinct states of *O. volvulus* infection (Soboslay et al., 1997). IgG4 responses were studied in *O. volvulus* microfilariae(Mf)-positive patients, in onchocerciasis patients with a post-patent *O. volvulus* infection (i.e Mf-negative but previously Mf-positive) and in infection-free endemic controls. OvAg-specific IgG4 responses were highest in Mf-positive patients (*p* < 0.01) and diminished with the post-patent state of *O. volvulus* infection (*p* < 0.01), but IgG4 responses remained elevated (*p* < 0.01) in post-patent cases when compared with Mf-negative endemic controls (those were never found *O. volvulus*-Mf-positive). In onchocerciasis patients who have received repeatedly ivermectin therapy for 16 years (Mai et al., 2007) we found that IgG4 responses lessened only moderately in Mf-negative ivermectin treated patients at 16 years post initial ivermectin, and their IgG4 reactivity remained significantly higher than in *O. volvulus* infection-free endemic controls. With an occult and expiring *O. volvulus* infection, IgG4 responses in onchocerciasis patients were similar as observed in endemic controls, and significantly lower (*p* < 0.0001) than in Mf-positive cases (Lechner et al., 2012). Those Mf-negative onchocerciasis-occult patients have previously been Mf-positive and became Mf-negative without having ever received treatment with ivermectin. This suggested that their infection expired gradually as their adult *O. volvulus* exceeded their natural life span (Lechner et al., 2012). Antibody responses to the *O. volvulus* adult worm extract (OvAg) and the recombinant antigen Ov16 were studied in Togo earlier, and the IgG4 sero-prevalence was 59% and 34%, respectively (Golden et al., 2016; Komlan et al., 2018). In Togo, the mass drug administration (MDA) of ivermectin (≥5 years, 150 μg/kg) has reduced the *O. volvulus* microfilarial prevalence markedly, but in the northern and central river basins the transmission of *O. volvulus* has never been interrupted completely (Yaméogo, 2008; WHO, 2014, WHO, 2015, WHO, 2016, WHO, 2017). *O. volvulus* infections still persisted in children and also in adults, and positive IgG4 responses indicated active *O. volvulus* infection (Komlan et al., 2018). Ivermectin may have a partial *in vivo* effect against infective third-stage larvae (L3) of *O. volvulus* but it has no effect against later larval stages (Taylor et al., 1988) and the standard dose of 150 μg/kg does not kill the adult O*. volvulus* and not disrupt embryogenesis or spermatogenesis (Awadzi et al., 1999). These observations have been confirmed recently (Pion et al., 2013), and onchocerciasis may persist at meso-endemic levels despite of >15 years of MDA with ivermectin (Kamga et al., 2016; Wanji et al., 2015). All three villages in the Region Centrale are situated in the river basin of Mô, and *O. volvulus* DNA was detected in *Simulium damnosum s.l.* in 2016 suggesting that parasite transmission continues (Komlan et al., 2018). With an ongoing low level parasite transmission, the endemic population will be exposed to trickle infections with L3 of *O. volvulus* and such exposure will stimulate specific antibody responses. In all tested age and gender groups the positive *O. volvulus* IgG4 responses and *M. perstans* real-time PCR results were similar. The infection prevalence was slightly higher in males (Brabin, 1990), positive responses enhanced with age and were higher in the Région Plateaux than Région Central suggesting that in the surveyed villages of the Plateaux region, despite repeated MDA with ivermectin and albendazole, favourable conditions for parasite transmission prevailed.

Positive IgG4 responses to *S. stercoralis* infective third-stage-larvae (SsL3Ag) were detected in up to 80% of the participants (Table 3). The ELISA we applied is based on *in vitro* cultured third-stage larvae of *S. strongyloides* which were used as the defined antigen for detection of IgG4 antibodies, but the accuracy of our in-house ELISA might not be the same as found for commercial tests. The diagnostic accuracy of five serologic tests for *S. stercoralis* infection was evaluated by Bisoffi et al. (2014), and their works showed that the detection of total IgG against somatic antigens of *S. stercoralis* larvae (IVD-ELISA) was sensitive with 88–97% (IC95%) and 96–99% (IC95%) specific. The *Strongyloides ratti* somatic antigen Bordier-ELISA showed 86–96% sensitivity and 91–97% specificity, the NIE-ELISA against recombinant L3 antigen 63–79% and 88–94%, the NIE-luciferase immuno-precipitation system 78–90% and 99–100%, and immune fluorescence against intact *S. stercoralis* filariform larvae was 91–99% sensitive and 83–91% specific. The accuracy and sensitivity of the *S. stercoralis* larval antigen-ELISA is amongst the highest, IgG4 subclass application may improve diagnosis (Rodrigues et al., 2007), and serology directed against larvae of *S. stercoralis* was found to be the best screening method in a *S. stercoralis* non-endemic setting (Buonfrate et al., 2017). However, when using the *S. stercoralis* larvae somatic antigen ELISA cross reactions with other parasites were observed for *Loa loa* (15%), *Wuchereria bancrofti* (13%), *Onchocerca volvulus* (10%), *Schistosoma* spp*.* (10%), and it was highest with 40% for hookworm and *Trichinella* spp. co-infections (Bisoffi et al., 2014). For sero-diagnosis of *S. stercoralis* the highest specific test is a luciferase immunoprecipitation system (LIPS) that employs a recombinant antigen (NIE), but this assay is not widely available (Krolewiecki et al., 2010, Bisoffi et al., 2014, Albonico et al., 2016). We are aware that *S. stercoralis*-specific PCR on stool samples will not demonstrate a particularly higher sensitivity in comparison to other coprological techniques, such as Baermann method and agar plate culture (Buonfrate et al., 2018). PCR would be appealing for its high specificity (Meurs et al., 2017) while its low sensitivity, when compared with serological tests, may be due to the irregular larval output observed in chronic strongyloidiasis (Buonfrate et al., 2018). PCR in combination with serology may increase the accuracy of epidemiological surveys on *S. stercoralis* infection prevalence. The positive IgG4 responses to *S. stercoralis* infective third-stage-larvae (SsL3Ag) in our study may be due to continuous exposure and re-infection, this may explain the differences between the two oldest and the youngest age group, but a continuous rise of IgG4 to SsL3Ag over age was not observed (Fig. 3, lower graph). Singly positive for SsL3Ag were 13.9%, and 15.7% of the participants tested IgG4-positive for SsL3Ag and also DNA-positive for *M. perstans* while being negative for OvAg-specific IgG4. Obvious again were the large differences between the regions for which regional differences of transmission of soil transmitted helminth parasites and also of *O. volvulus* may account. Furthermore, the SsL3Ag-specific IgG4 responses may either link with the intensity of infection, as described for schistosomiasis (van Dam et al., 1996), but may also reflect repeated *S. stercoralis* auto-infection with L1 larvae which may stimulate specific antibody production; this may occur even several years after exposure, and strongyloidiasis may persist lifelong. Repeated ivermectin and albendazole distribution in our study area will effect on prevalence of *S. stercoralis* larvae in stool samples and on serology (Anselmi et al., 2015). Strongyloidiasis appears particularly overlooked (Albonico et al., 2016), although endemic in many countries, and large-scale preventive chemotherapy with ivermectin significantly reduced and maintained low the prevalence of *S. strongyloides* in Ecuador and Tanzania (Anselmi et al., 2015; Barda et al., 2017). Repeated follow ups of populations who receive MDA with ivermectin and albendazole may show to which extent serological conversion to *S. stercoralis* may occur, and our present surveys on *S. stercoralis* were the first ones conducted in that regions.

## 5 Conclucion

Mansonelliasis, onchocerciasis and strongyloidiasis remain prevalent in the surveyed regions, yet with local differences, and their levels of infection have been affected to a certain degree by MDA with ivermectin and albendazole. Our observations suggest that transmission of *M. perstans*, *O. volvulus* and *S. stercoralis* may be ongoing. The degree of positive test results in the examined rural communities advocate for the continuation of MDA with ivermectin and albendazole and for bi-annual therapies which should further reduce helminth infection levels and interrupt parasite transmission. Further extended regional surveys are recommendable which specifically address the morbidity and the progress towards elimination of *M. perstans, O. volvulus* and *S. stercoralis* infections.

## Conflicts of interest

None.
